# Roles of Ceramide Glucosyltransferase in Controlling Cerebral Microvascular Endothelial Integrity and Angiogenesis Post-Ischemia/Reperfusion

**DOI:** 10.1007/s12975-026-01488-9

**Published:** 2026-08-21

**Authors:** Ayan Mohamud Yusuf, Maria Zafar, Nina Hagemann, Ulf Brockmeier, Fabian Schumacher, Burkhard Kleuser, Tobias Tertel, Bernd Giebel, Erich Gulbins, Dirk M. Hermann

**Affiliations:** 1 Department of Neurology, University Hospital Essen, University of Duisburg-Essen, Hufelandstr. 55, 45122 Essen, Germany; 2 Department of Pharmacology and Toxicology, Institute of Pharmacy, Freie Universität Berlin, Berlin, Germany; 3 Institute of Transfusion Medicine, University Hospital Essen, University of Duisburg-Essen, Essen, Germany; 4 Institute of Molecular Biology, University Hospital Essen, University of Duisburg-Essen, Essen, Germany

**Keywords:** Ischemic stroke, Glycosphingolipids, Exosomes, Microvascular remodeling, Light-sheet microscopy, Middle cerebral artery occlusion

## Abstract

Sphingolipids critically regulate microvascular integrity and function, but the role of glycosphingolipids in endothelial survival and angiogenesis remains poorly defined. Herein, we experimentally deactivated or activated UDP-glucose ceramide glucosyltransferase (UGCG), which converts ceramide to glucosylceramide, by the pharmacological inhibitor D-threo-1-phenyl-2-decanoylamino-3-morpholino-1-propanol (D-PDMP), siRNA-mediated knockdown or the pharmacological activator L-PDMP. Sphingolipid and glycosphingolipid profiles were examined by liquid chromatography-tandem-mass spectrometry. Effects on endothelial survival, proliferation, transwell migration, extracellular vesicle (EV) release and tube formation were assessed in human cerebral microvascular endothelial cells (hCMEC/D3). *In vitro*, pharmacological UGCG deactivation near-completely suppressed hexosylceramide levels and at high dose increased ceramide and sphingosine-1-phosphate (S1P), a known pro-angiogenic sphingolipid, while UGCG knockdown moderately decreased mostly short (C16, C18) hexosylceramides. UGCG activation increased hexosylceramide without significantly altering ceramide and S1P. Pharmacological UGCG deactivation increased endothelial tube formation, a marker of angiogenesis, but at high dose decreased endothelial survival, whereas UGCG knockdown and UGCG activation reduced endothelial tube formation and migration or proliferation, respectively. Pharmacological UGCG deactivation and activation, but not UGCG knockdown increased endothelial release of EVs with anti-angiogenic activity. In mice exposed to transient middle cerebral artery occlusion, pharmacological UGCG deactivation and activation reduced the length and branch density of small-sized (< 4 µm) and intermediate (4–5.4 µm) cerebral microvessels in the reperfused striatum as revealed by 3D light-sheet microscopy, indicative of microvascular endothelial degeneration. Our results suggest that pharmacological UGCG deactivation promotes angiogenesis *in vitro* probably via S1P elevation. *In vivo*, UGCG deactivation failed to stabilize microvascular network integrity post-ischemia/reperfusion, presumably due to ceramide-associated cell stress.

**Supplementary Information:**

The online version contains supplementary material available at https://doi.org/10.1007/s12975-026-01488-9.

## Introduction

Sphingolipids are important regulators of vascular integrity and function. Among these, ceramide and sphingosine-1-phosphate (S1P) are particularly well-studied due to their opposing roles in endothelial survival and vascular remodeling [3, 29]. Ceramide is known to promote apoptotic signaling and inhibit cell proliferation, whereas S1P increases endothelial survival, migration, and angiogenesis [26, 43]. The balance between these sphingolipids is tightly controlled by enzymes of the sphingolipid pathway and plays a key role in regulating microvascular integrity and angiogenesis in response to cerebral ischemia/reperfusion (I/R) [30, 31]. Ceramide contributes to the organization of membrane microdomains and serves as a platform for the assembly of signaling complexes that control key cellular processes such as apoptosis and growth arrest [39]. Ceramide also controls membrane fluidity and fusogenicity and thereby regulates crucial cell physiological processes such as endosomal uptake and release of extracellular vesicles (EVs) [31, 42]. Upon I/R injury, ceramide levels rapidly rise rapidly due to the activation of sphingomyelinases (most notably acid sphingomyelinase) hydrolyzing sphingomyelin into ceramide [31]. In ischemic stroke, ceramide strongly accumulates in cerebral microvessels, promoting endothelial dysfunction and cell damage [31]. Clinically, elevated ceramide concentrations in the plasma of ischemic stroke patients are associated with unfavorable stroke outcome [9], and distinct plasma ceramide ratios have been shown to predict cardiovascular death in patients with stable coronary artery disease and acute coronary syndrome independent of established lipid markers and C-reactive protein in three large European cohorts [25]. These findings support the recognition of ceramides as mechanistically and prognostically relevant lipids in vascular pathologies. Brain recovery post-I/R critically depends on the restoration of cerebral microvascular networks through processes involving endothelial protection and angiogenesis [2, 24]. Thus, understanding the impact of sphingolipids on post-ischemic vascular remodeling is essential for developing strategies that enhance brain repair and ultimately allow improving stroke outcome [15].

Compared with ceramide, the role of glycosylated ceramides is much less examined in cerebral I/R injury. UDP-glucose ceramide glucosyltransferase (UGCG) catalyzes the first glycosylation step in the biosynthesis of glycosphingolipids by converting ceramide to glucosylceramide, the precursor of more complex glycosphingolipids. Phenyl-2-decanoylamino-3-morpholino-1-propanol (PDMP) is widely used to pharmacologically manipulate glucosylceramide formation and to investigate its impact on cellular processes [5]. PDMP exists in two stereoisomers: D-threo-1-phenyl-2-decanoylamino-3-morpholino-1-propanol (D-PDMP), an UGCG inhibitor, and L-threo-1-phenyl-2-decanoylamino-3-morpholino-1-propanol (L-PDMP), an UGCG activator [5]. In neuroepithelioma cells, UGCG deactivation by D-PDMP reduced glucosylceramide levels and increased the cellular susceptibility to apoptotic injury [38], indicating that glycosylated ceramide is neuroprotective. In cortical neurons, UGCG inhibition by D-PDMP induced neurite retraction and impaired neuronal viability [45]. UGCG inhibition by D-PDMP inhibited neuronal plasma membrane Ca^2^⁺-ATPase (PMCA), which maintains intracellular calcium homeostasis [21]. In a rat model of focal cerebral ischemia induced by permanent middle cerebral artery occlusion (MCAO), the intraperitoneally administered UGCG activator L-PDMP increased glucosylceramide levels in the ischemic cortex and improved learning and memory [17]. In a rat model of global cerebral ischemia induced by 4-vessel occlusion, intraperitoneally administered L-PDMP reduced post-ischemic memory deficits, while D-PDMP had no effect [20]. In both studies, L-PDMP and D-DPMP were administered in the post-acute phase, starting 24 h post-I/R [17, 20]. *In vitro*, the UGCG activator L-PDMP increased spontaneous synchronous oscillations of cortical neurons indicative of functional synapse formation, whereas the UGCG inhibitor D-PDMP had opposite effects [20]. Together, these findings point towards a neuronal survival and plasticity-promoting role of glucosylceramide in cerebral I/R injury. While glycosphingolipid metabolism has been investigated in neuronal function, its role in cerebral endothelial biology remains poorly understood. In venous malformation, increased UGCG expression was observed in affected vessels and endothelial cells, and UGCG modulation altered endothelial responses through mechanisms involving AKT/mTOR signaling [6]. However, whether UGCG-dependent glycosphingolipid regulation influences endothelial responses during cerebral ischemia remains unknown. Since ceramide is strongly enriched in cerebral microvessels post-MCAO [31], we asked how UGCG deactivation and activation influences microvascular network integrity and angiogenesis following I/R. To elucidate this question, we investigated the effects of pharmacological UGCG deactivation by D-PDMP, siRNA-mediated UGCG knockdown and pharmacological UGCG activation by L-PDMP on sphingolipid and glycosphingolipid profiles. We examined resulting effects on endothelial survival and angiogenesis in human cerebral microvascular endothelial cells (hCMEC/D3) *in vitro* and assessed impacts on cerebral microvascular network integrity and remodeling in a mouse model of transient MCAO. In view of the neuronal survival and plasticity promoting effects of UGCG activation [17, 20], we hypothesized that UGCG activation should similarly enhance cerebral microvascular integrity and angiogenesis following I/R injury.

## Methods

### Legal Issues and Animal Housing

All experiments were performed with permission of the Landesamt fuer Natur, Umwelt und Verbraucherschutz (LANUV), which is part of the Ministry for Environment, Agriculture, Conservation and Consumer Protection (MULNV) of the State of North Rhine-Westphalia, in accordance with EU guidelines (Directive 2010/63/EU) for the care and use of laboratory animals. Animal experiments were reported according to ARRIVE guidelines. Mice were kept in a regular inverse 12 h:12 h light/dark cycle in groups of 5 animals/cage and had free access to food and drinking water. As exclusion criteria, mice were removed from the study if suffering from respiratory abnormalities or from severe nurturing handicaps resulting in a weight loss > 20%.

### Middle Cerebral Artery Occlusion

Focal cerebral ischemia was induced in male C57BL/6j mice (10–12 weeks, Envigo, Horst, Netherlands). Mice were anesthetized with 1.0–1.5% isoflurane (30% O_2_, remainder N_2_O). Rectal temperature was maintained between 36.5 and 37.0 °C using a feedback-controlled heating system. Cerebral blood flow was recorded by laser Doppler flow (LDF) measurement using a flexible 0.5 mm fiber-optic probe (Perimed, Rommerskirchen, Germany) attached to the intact skull overlying the MCA territory (2 mm posterior, 6 mm lateral from bregma). For MCAO, a midline neck incision was made and the left common and external carotid arteries were isolated and ligated. The internal carotid artery (ICA) was temporally clipped. A silicon-coated monofilament was introduced via a small incision into the common carotid artery (CCA) and advanced to the carotid bifurcation for MCAO. Reperfusion was initiated after 30 min by monofilament removal. Wounds were carefully sutured. The opioid buprenorphine (0.1 mg/kg; Temgesic, Essex Pharma, Munich, Germany) was subcutaneously administered before surgery, and the antiphlogistic carprofen (5 mg/kg; b.i.d., Bayer Vital, Leverkusen, Germany) was subcutaneously administered for 3 days after surgery. To ensure unbiased group allocation, mice were randomly assigned to treatment groups using a computer-generated random number sequence before the experiment began. Strict randomization procedures were maintained throughout the experimental process. The researcher responsible for conducting the animal experiments (AMY) was fully blinded at all stages by another researcher (NH), who prepared the vehicle, D-PDMP (Cayman Chemical, Cat. No. 10178, Michigan, U.S.A.) and L-PDMP (Abcam, Cat. No. ab144046, Cambridge, U.K.) solutions. These solutions were labeled as Solution A, Solution B, and Solution C and their identities were disclosed only after the study had been completed. Vehicle, D-PDMP (40 mg/kg) or L-PDMP (40 mg/kg) were intraperitoneally injected starting one day after MCAO until fourteen days after MCAO as previously reported [20].

### FITC-Albumin Perfusion and Light-Sheet Microscopy

Mice were deeply anesthetized and transcardially perfused with 4% paraformaldehyde, followed by infusion of 10 ml FITC-albumin hydrogel (Sigma-Aldrich, Cat. No. A9771 Deisenhofen, Germany), as previously reported [10, 11]. Brains were subsequently removed, post-fixed overnight at 4 °C in 4% PFA. After brain dehydration and clearing using a tetrahydrofuran gradient (Sigma-Aldrich, Cat. No. 34865) and ethyl cinnamate (Sigma-Aldrich, Cat. No. 112372), brains were scanned at 2 μm steps using an UltraMicroscope Blaze (LaVision BioTec, Bielefeld, Germany) light sheet microscope using a 488-nm diode laser and a 525/50-nm band-pass emission filter. Regions of interest measuring 508 × 508 × 1000 µm were chosen in the dorsolateral striatum, which is the core of the middle cerebral artery territory. Autofluorescence was captured with a 561-nm laser and detected via a 595/40-nm band-pass emission filter.

### Analysis of Microvascular Networks in the Reperfused Ischemic Brain

The microvascular network analysis pipeline VesselExpress was applied to analyze brain vasculature in FITC-albumin perfused brains as described before [37]. The pipeline utilizes a dual Frangi filter strategy for vessel segmentation, which allows analyzing diameters of small, intermediate and large microvessels with high accuracy and unprecedented speed. After segmentation of raw images, vessel centerlines were extracted from the binary images using a parallel thinning algorithm from the scikit-image Python package. These centerlines were then converted into undirected graphs via the 3scan Python toolkit. A depth-first search (DFS) traced the vessels, and vascular parameters, including lengths and branch numbers, were extracted as before [10, 11]. FITC fluorescence was quantified in Fiji/ImageJ. To quantify FITC-albumin extravasation, total FITC fluorescence was measured within the region of interest. Intravascular FITC was segmented using fluorescence intensity thresholding, and extravascular FITC was calculated by subtracting the intravascular FITC signal from the total FITC signal. Values were normalized to vessel area and expressed as arbitrary units (a.u.).

### Cerebral Endothelial Cell Culture

Immortalized human cerebral microvascular endothelial cells (hCMEC/D3) were cultured in endothelial basal medium (EBM-2, Lonza, Cat. No. CC-3156, Basel, Switzerland) supplemented with 5% fetal bovine serum (FBS, Cat. No. A5670701, Thermo Fisher Scientific, MA, U.S.A.), 100 U/ml penicillin/streptomycin (Thermo Fisher Scientific, Cat. No. 15140122), 1.4 µM hydrocortisone (Sigma-Aldrich, Cat. No H0888), 5 µg/ml ascorbic acid (Sigma-Aldrich, Cat. No. A4544), 1% chemically defined lipid concentrate (Thermo Fisher Scientific, Cat. No. 11905031) and 10 mM HEPES (Thermo Fisher Scientific, Cat. No. 15630056).

### Small Interfering RNA (siRNA) Knockdown

Human UDP-glucose ceramide glucosyltransferase (UGCG) knockdown *in vitro* was induced by small interfering RNA (siRNA). Cells were transfected with human UGCG siRNA-SMARTpool (L-006441–00–0005, Horizon Discovery, Cambridge, U.K.) or scrambled siRNA (D-001810–10-05, Horizon Discovery) as a control. Transfections were performed according to the manufacturer’s instructions using Dharmafect transfection reagents.

### Endothelial Survival Assay

2 × 10^4^ cells were seeded into 96-well plates and treated with vehicle, D-PDMP (25 µM or 50 µM) or L-PDMP (25 µM or 50 µM). D-PDMP and L-PDMP solutions were obtained by diluting 10 mM stock solutions (in ethanol) in endothelial medium to achieve the final concentrations. After 24 h, cells were incubated with 0.5 mg/ml 3-(4,5-dimethyl-2-thiazolyl)−2,5-diphenyl-2H-tetrazolium bromide (MTT; Biomol, Cat. No. 15655, Hamburg, Germany) for 2 h. Formazon formation was measured at 570 nm on a microplate reader (iMark Detection; Bio-Rad Laboratories, Hercules, CA, U.S.A.). Samples were analyzed in triplicates of which mean values were calculated.

### Endothelial Proliferation Assay

3 × 10^5^ cells were seeded per well of a 6-well plate in low serum medium (1.25% FBS) and treated with vehicle, D-PDMP (25 µM or 50 µM, as above) or L-PDMP (25 µM or 50 µM, as above). After 72 h, the cells were trypsinized and 10 µl of the cell suspension was mixed with 10 µl of 0.4% trypan blue. 10 µl of this sample mixture was loaded on EVE™ cell counting slides (NanoEnTek, Seoul, South Korea) and cell numbers were quantified using an EVE™ automated cell counter (NanoEnTek). Samples were analyzed in triplicates, of which mean values were formed.

### Endothelial Transwell Migration Assay

Cell migration was assessed using a modified Boyden chamber. 3 × 10^4^ cells were seeded in the upper compartment of polycarbonate membrane inserts (8.0 μm pores) that contained serum-reduced (1.25% FBS) medium. Vehicle, D-PDMP (25 µM or 50 µM, as above) or L-PDMP (25 µM or 50 µM, as above) were administered into the lower compartment that contained 5% FBS. Cells that did not migrate were removed after 24 h. Migrated cells were fixed with 4% PFA and stained with Hoechst 33342 (Thermo Fisher Scientific, Cat. No. 62249, U.S.A.). Photomicrographs were taken with a 20 × objective using the EVOS digital inverted microscope (Advanced Microscopy Group). Experiments were done in duplicates, for which mean values were formed.

### Endothelial Tube Formation Assay

To evaluate the formation of capillary-like tubular structures, 60 µl matrigel (Corning, NY, U.S.A.) were pipetted into 96-well plates. The gel solidified at 37 °C for 30 min. 3 × 10^4^ cells were seeded and treated with vehicle, D-PDMP (25 µM or 50 µM, as above), L-PDMP (25 µM or 50 µM, as above) or EVs at a concentration of 25 µg/ml or particle numbers of 1 × 10^8^ particles/well. After 20 h, photomicrographs were taken with a 2 × objective using the EVOS digital inverted microscope (Advanced Microscopy Group, Bothell, WA, U.S.A.). Closed tubes, microvascular length, branching point numbers and numbers of branches were determined using Image J (NIH). Experiments were done in triplicates, for which mean values were calculated.

### Real-Time Quantitative Polymerase Chain Reaction (RT-qPCR)

RNA was isolated according to the phase extraction method using TRIzol (Thermo Fisher Scientific, Cat. No. 15596026)/chloroform (Sigma-Aldrich, Cat. No. 319988) and treated with DNase I (Thermo Fisher Scientific, Cat. No. 18068015). Complementary DNA (cDNA) was generated by reverse transcription with SuperScript II (Thermo Fisher Scientific, Cat. No. 18064071) using oligo(dt) primers and random hexamers. Real-time quantitative polymerase chain reaction (RT-qPCR) was performed with SYBR-Green (Thermo Fisher Scientific, Cat. No. 4385612) in a StepOnePlus real-time PCR system with two different primers targeting UDP-glucose ceramide glucosyltransferase (Merck, Darmstadt, Germany) and β-actin (Biomol, Hamburg, Germany) as a housekeeping gene. The following primers were used:

**Table:** 

UDP-glucose ceramide glucosyltransferase	Primer 1: Forward 5´-TTCATGTGTCATTGCCTGGC-3´; Reverse 5´- AGCGTAATCTGTAGCGACCA-3´ Primer 2: Forward 5´- GACCTGGCCTTGGAGGGAAT-3´; Reverse 5´-GAGACACCTGGGAGCTTGCT-3´
β-actin	Forward 5´-GGACTTCGAGCAAGAGATGG-3´; Reverse 5´- AGCACTGTGTTGGCGTACAG-3´

Results were quantified using the 2 − ∆∆Ct method. Samples were analyzed in triplicates of which mean values were formed

### Liquid Chromatography Tandem–Mass Spectrometry

Cell suspensions were subjected to lipid extraction using 1.5 ml methanol/chloroform (2:1, v:v) as described [34]. The extraction solvent contained d_7_-sphingosine-1-phosphate (d_7_-S1P), C17 ceramide (C17 Cer), d_31_-C16 sphingomyelin (d_31_-C16 SM), C17 glucosyl(β) ceramide (C17 HexCer) and C17 lactosyl(β) ceramide (C17 LacCer) (all Avanti Polar Lipids, Alabaster, USA) as internal standards. Chromatographic separations were achieved on a 1290 Infinity II HPLC (Agilent Technologies, Waldbronn, Germany) equipped with a Poroshell 120 EC-C8 column (3.0 × 150 mm, 2.7 µm,Agilent Technologies). MS/MS analyses were carried out using a 6495 C triple-quadrupole mass spectrometer (Agilent Technologies) operating in the positive electrospray ionization mode (ESI +). The following mass transitions were recorded (qualifier product ions in parentheses): *m/z* 380.3 → 264.3 (82.1) for S1P, and *m/z* 387.3 → 271.3 (82.1) for d_7_-S1P; ceramides: *m/z* 520.5 → 264.3 (282.3) for C16 Cer, *m/z* 534.5 → 264.3 (282.3) for C17 Cer, *m/z* 548.5 → 264.3 (282.3) for C18 Cer, *m/z* 576.6 → 264.3 (282.3) for C20 Cer, *m/z* 604.6 → 264.3 (282.3) for C22 Cer, *m/z* 630.6 → 264.3 (282.3) for C24:1 Cer, and *m/z* 632.6 → 264.3 (282.3) for C24 Cer; sphingomyelins: *m/z* 703.6 → 184.1 (86.1) for C16 SM, *m/z* 731.6 → 184.1 (86.1) for C18 SM, *m/z* 734.6 → 184.1 (86.1) for d_31_-C16 SM, *m/z* 759.6 → 184.1 (86.1) for C20 SM, *m/z* 787.7 → 184.1 (86.1) for C22 SM, *m/z* 813.7 → 184.1 (86.1) for C24:1 SM, and *m/z* 815.7 → 184.1 (86.1) for C24 SM; hexosylceramides: *m/z* 700.6 → 264.3 (682.6) for C16 HexCer, *m/z* 714.6 → 264.3 (696.6) for C17 HexCer, *m/z* 728.6 → 264.3 (710.6) for C18 HexCer, *m/z* 756.6 → 264.3 (738.6) for C20 HexCer, *m/z* 784.6 → 264.3 (766.6) for C22 HexCer, *m/z* 810.7 → 264.3 (792.7) for C24:1 HexCer, and *m/z* 812.7 → 264.3 (794.7) for C24 HexCer; lactosylceramides: *m/z* 862.6 → 264.3 (520.5) for C16 LacCer, *m/z* 876.6 → 264.3 (534.5) for C17 LacCer, *m/z* 890.6 → 264.3 (548.5) for C18 LacCer, *m/z* 918.6 → 264.3 (576.6) for C20 LacCer, *m/z* 946.6 → 264.3 (604.6) for C22 LacCer, *m/z* 972.7 → 264.3 (630.7) for C24:1 LacCer, and *m/z* 974.7 → 264.3 (632.6) for C24 LacCer. Peak areas of Cer, SM, HexCer and LacCer subspecies, as determined with MassHunter software (version 10.1, Agilent Technologies), were normalized to those of their corresponding internal standards (C17 Cer, d_31_-C16 SM, C17 HexCer or C17 LacCer, respectively) followed by external calibration in the range of 10 fmol to 15 pmol (HexCer and LacCer) or 50 pmol (Cer and SM) on column. S1P was directly quantified via its deuterated internal standard d_7_-S1P (0.125 pmol on column). Determined sphingolipid amounts were normalized to cell count.

### Isolation and Analysis of Small Extracellular Vesicles (EVs)

To prepare small EVs, a well-established PEG-ultracentrifugation protocol was applied [28]. Briefly, conditioned cell culture media were collected and centrifuged at 2,000 g for 15 min at 4 °C, followed by centrifugation at 10,000 g for 45 min at 4 °C (5810R centrifuge, Eppendorf, Hamburg, Germany). Supernatants were filtered through a 0.22 µm filter (Sartorius, Göttingen, Germany) and supplemented with 75 mM NaCl and 10% polyethylene glycol-6000 (PEG,Cat. No. 8.07491, Sigma-Aldrich). Following incubation over night at 4 °C, small EVs were concentrated at 1,500 g for 30 min at 4 °C (Avanti J-26 XP centrifuge, Beckmann Coulter, Brea, CA, U.S.A.). Pellets were then resuspended in 0.9% NaCl (Sigma-Aldrich, Cat. No. S9888), transferred to ultra-clear centrifuge bottles (Beckmann Coulter) and pelleted by ultracentrifugation at 110,000 g for 130 min at 4 °C (Optima L7-65, k factor: 133, Beckmann Coulter). EV pellets were resuspended in 0.9% NaCl supplemented with 10 mM HEPES (Thermo Fisher Scientific, Cat. No. 15630056) and stored in low retention microcentrifuge tubes (Kisker Biotech, Cat. No. G017, Steinfurt, Germany). EV sizes were analyzed by nanoparticle tracking analysis (NTA; Particle Metrix, Meerbusch, Germany), as described previously [36]. Protein contents were determined by conventional BCA assays conducted according to the vendor’s recommendation. For semiquantitative EV analysis, EVs were labeled with anti-CD9–FITC (MEM-61, Exbio, Vestec, Czech Republic) and anti-CD63–APC (MEM-259, Exbio) antibodies and analyzed on the ImageStreamX MkII flow imager (Cytek, CA, U.S.A.) using our optimized protocols [8, 41].

### Statistical Analysis

Data were analyzed by one-way or two-way ANOVA, as adequate, followed by least significant difference (LSD) posthoc tests. For comparisons between two groups, unpaired two-tailed t tests were used. Data distribution was assessed using the Shapiro–Wilk test. P values < 0.05 were defined to indicate statistical significance. The statistical details (n numbers and tests) are given in the figure legends. Statistical analyses were performed using GraphPad Prism version 9.5.1 software. Data are expressed as mean + standard deviation (SD).

## Results

### Pharmacological UGCG Deactivation Decreases Hexosylceramide Levels and at a High Dose Increases Ceramide and S1P Levels in Cerebral Endothelial Cells

**Fig. 1:**
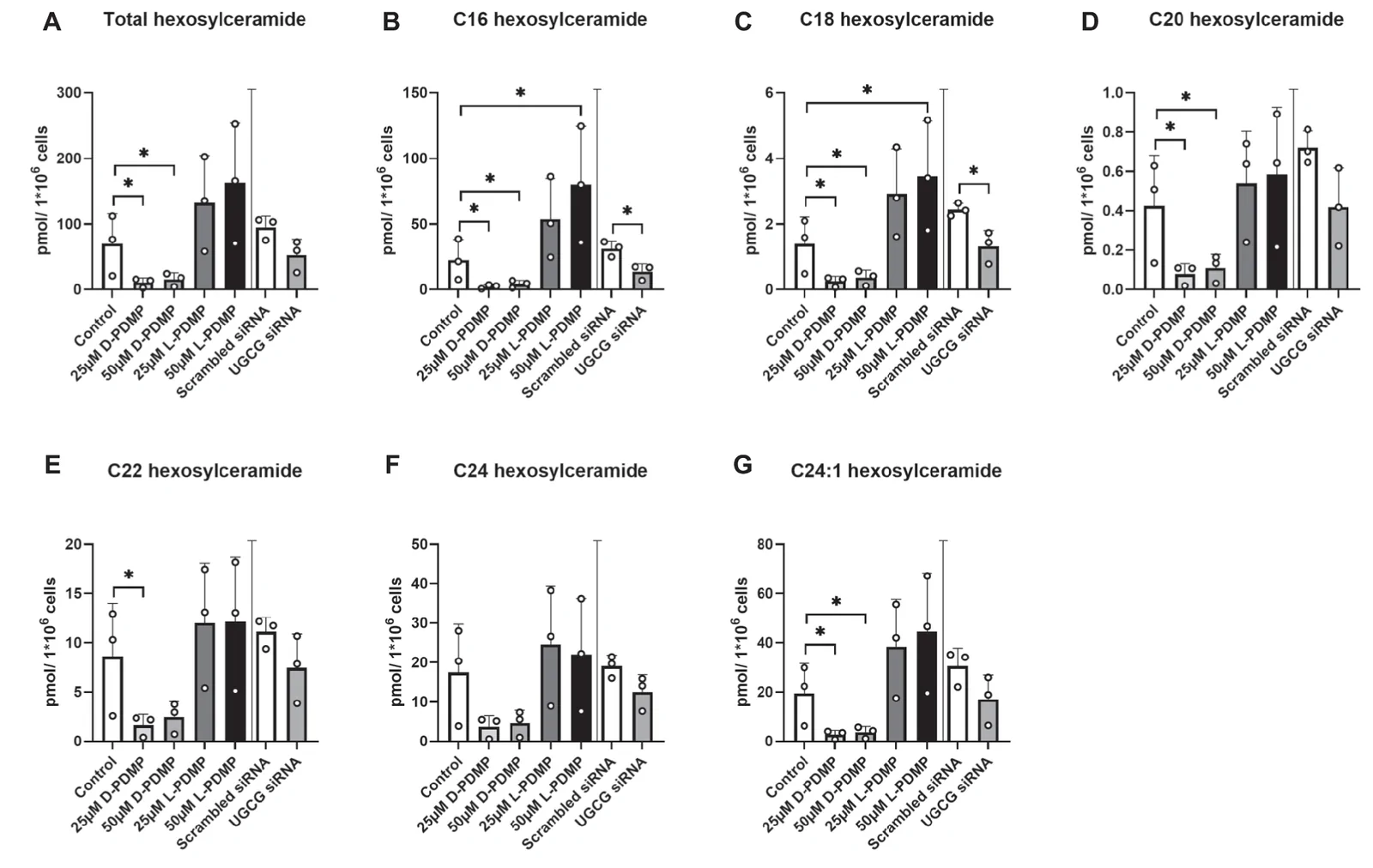
Pharmacological UGCG deactivation and UGCG knockdown decrease hexosylceramide levels, while UGCG activation increases hexosylceramide levels. hCMEC/D3 were treated with vehicle, D-PDMP or L-PDMP (25 µM or 50 µM each) for 24 h, or were transfected with scrambled siRNA or UGCG siRNA, followed by mass spectrometric profiling of total hexosylceramide (in **A**) and individual hexosylceramide species (in **B-G**). Results are expressed as pmol per 1 million cells. Data are presented as mean + SD. Statistical analysis was performed using one-way ANOVA followed by LSD post hoc tests for the pharmacological UGCG deactivation and activation experiments, or two-tailed unpaired t-tests for the siRNA mediated knockdown experiments. Statistical significance is indicated as *p < 0.05 (*n* = 3 independent samples/group)

**Fig. 2:**
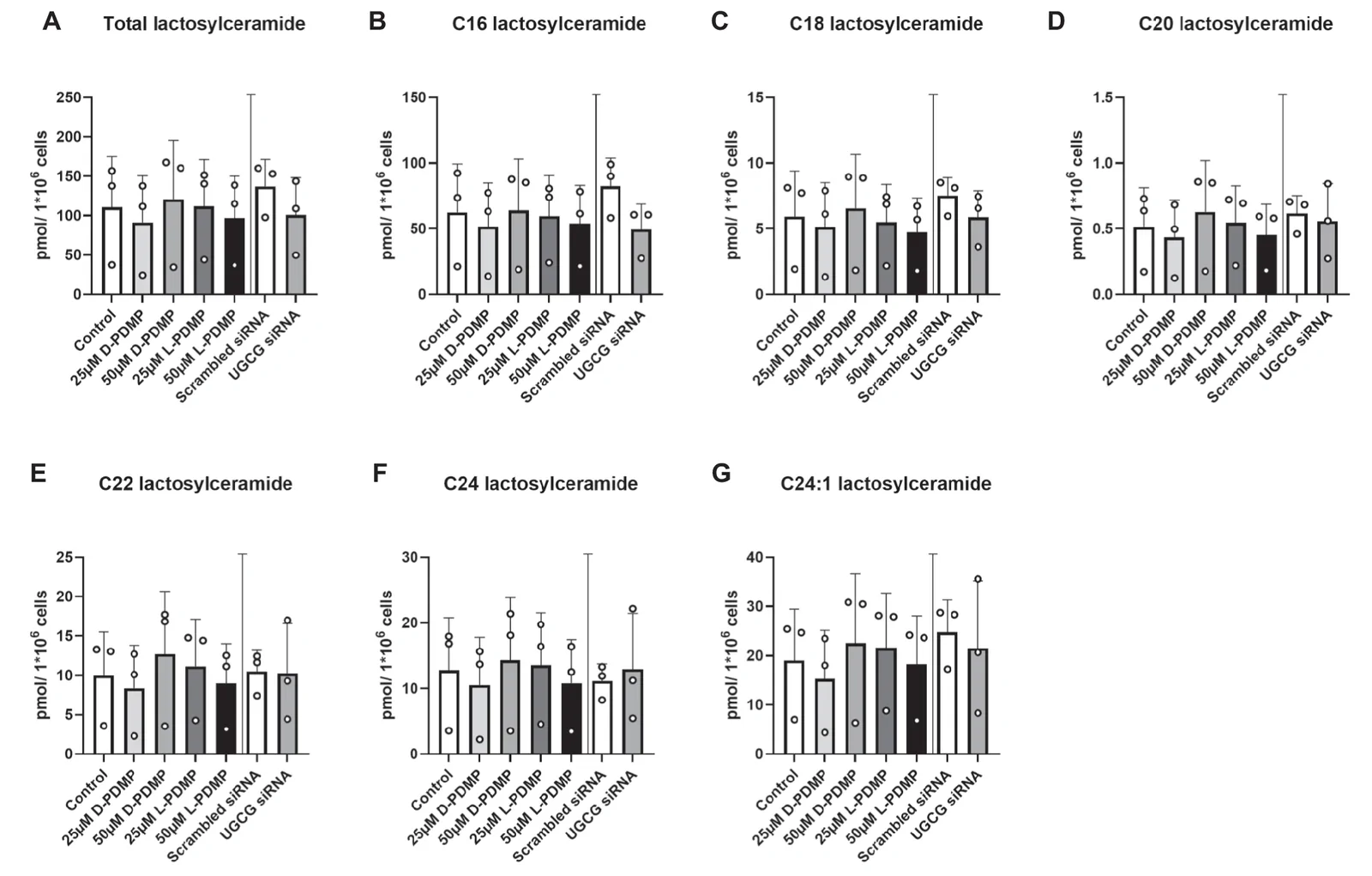
Pharmacological UGCG modulation and UGCG knockdown do not influence lactosylceramide levels. hCMEC/D3 were treated with vehicle, D-PDMP or L-PDMP (25 µM or 50 µM each) for 24 h, or were transfected with scrambled siRNA or UGCG siRNA, followed by mass spectrometric profiling of total lactosylceramide (**A**) and individual lactosylceramide species (**B-G**). Results are expressed as pmol per 1 million cells. Data are presented as mean + SD. Statistical analysis was performed using one-way ANOVA followed by LSD post hoc tests for the pharmacological UGCG deactivation and activation experiments, or two-tailed unpaired t-tests for the siRNA mediated knockdown experiments. No significant group differences were found (*n* = 3 independent samples/group)

**Fig. 3:**
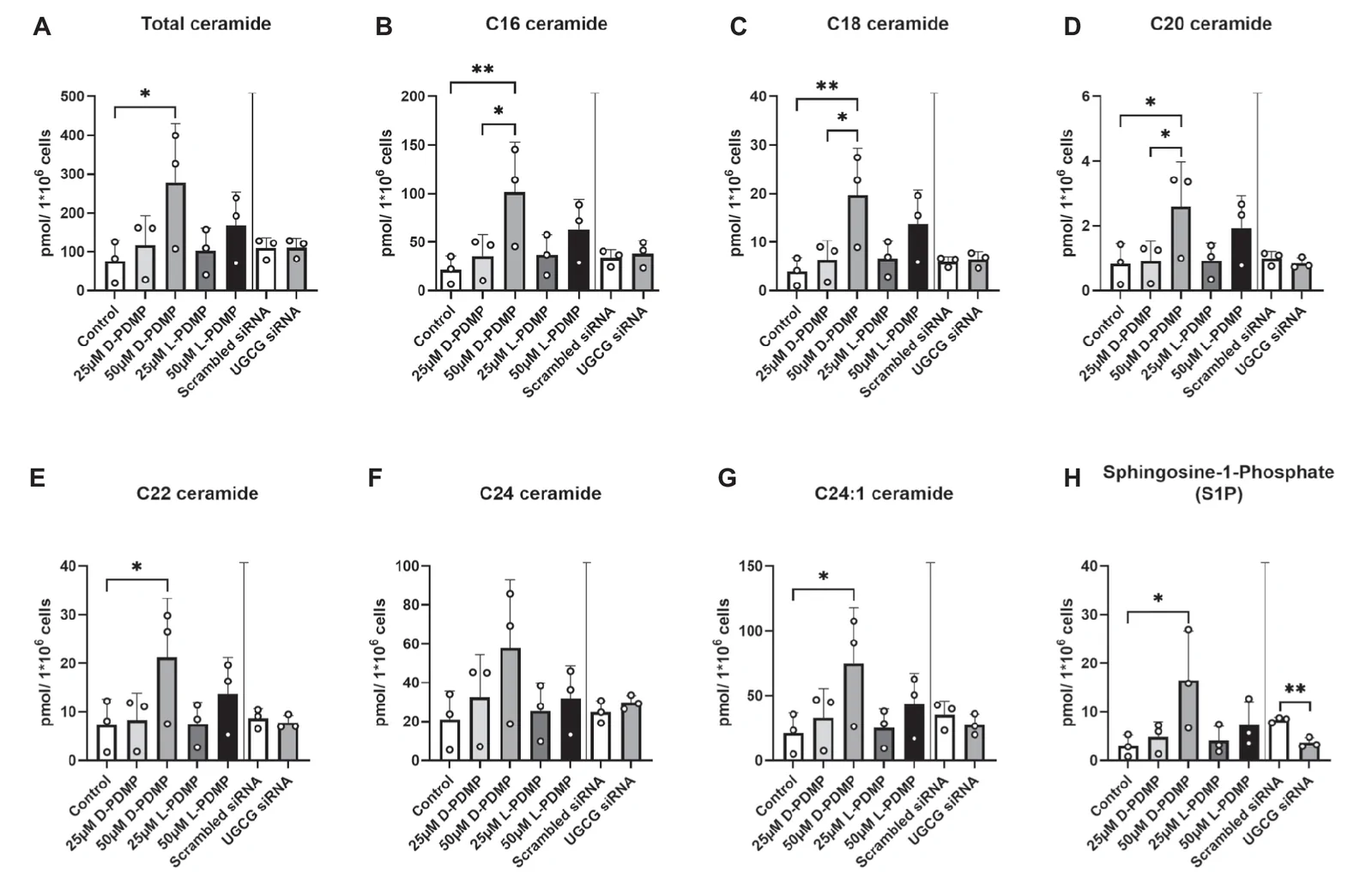
Pharmacological UGCG deactivation, but not activation significantly increases ceramide and S1P levels, while siRNA mediated UGCG knockdown does not influence ceramide levels, but reduces S1P level. Human cerebral microvascular endothelial cells (hCMEC/D3) were treated with vehicle, D-PDMP or L-PDMP (25 µM or 50 µM each) for 24 h, or were transfected with scrambled siRNA or UGCG siRNA, followed by mass spectrometric profiling of total ceramide (**A**), individual ceramide species (**B-G**) and sphingosine-1-phosphate (S1P) (**H**). Results are expressed as pmol per 1 million cells. Data are presented as mean + SD. Statistical analysis was performed using one-way ANOVA followed by LSD post hoc tests for the pharmacological UGCG deactivation and activation experiments, or two-tailed unpaired t-tests for the siRNA mediated knockdown experiments. Statistical significance is indicated as *p < 0.05 or **p < 0.01 (*n* = 3 independent samples/group)

**Fig. 4:**
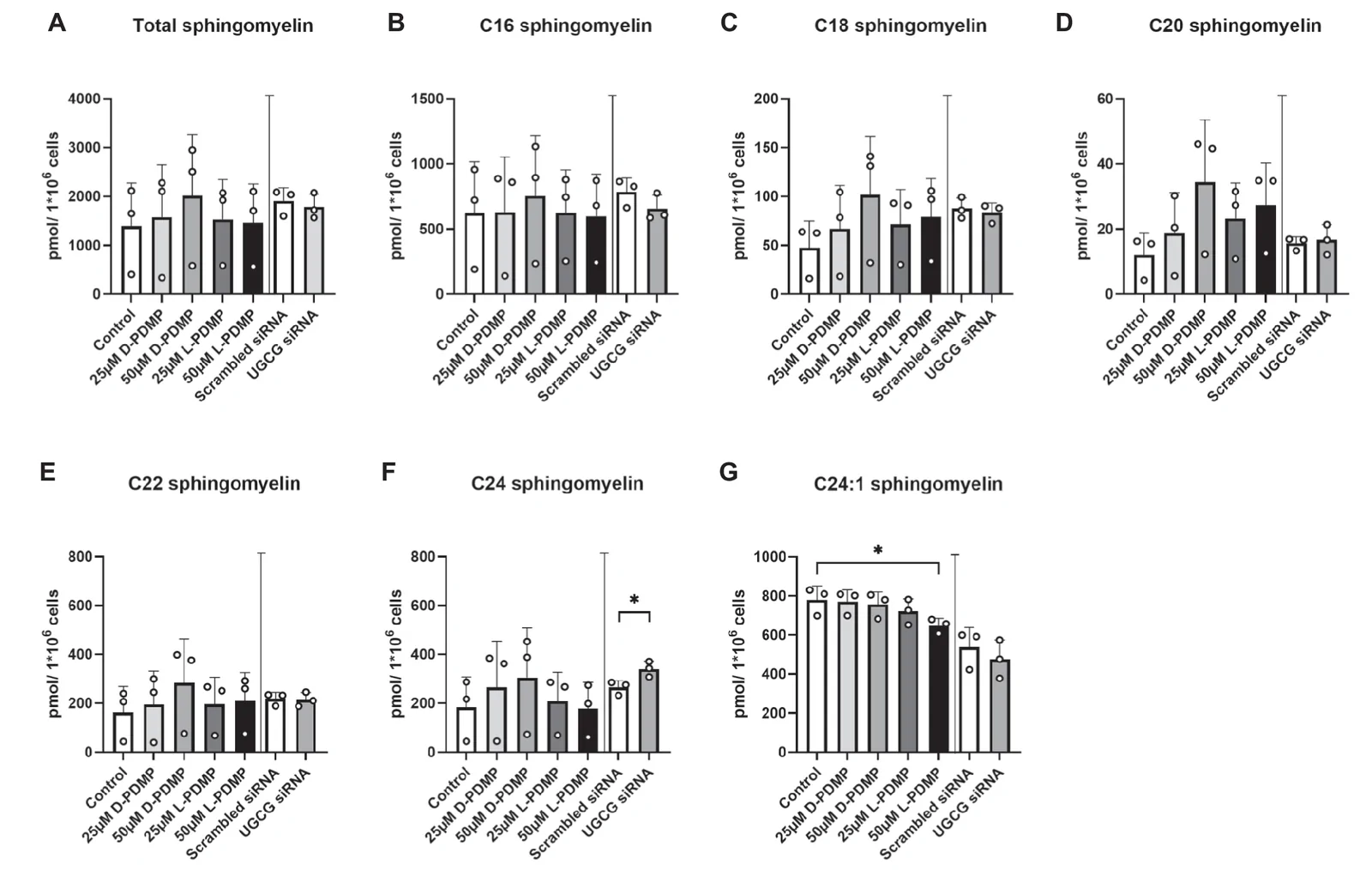
Sphingomyelin levels are largely unaffected by UGCG modulation, with the exception of a decrease in C24:1 sphingomyelin upon UGCG activation and an increase in C24 sphingomyelin upon UGCG knockdown. hCMEC/D3 were treated with vehicle, D-PDMP or L-PDMP (25 µM or 50 µM each) for 24 h, or were transfected with scrambled siRNA or UGCG siRNA, followed by mass spectrometric analysis of total sphingomyelin (**A**) and individual sphingomyelin species (**B-G**). Results are expressed as pmol per 1 million cells. Data are presented as mean + SD. Statistical analysis was performed using one-way ANOVA followed by LSD post hoc tests for the pharmacological UGCG deactivation and activation experiments, or two-tailed unpaired t-tests for the siRNA mediated knockdown experiments. Statistical significance is indicated as *p < 0.05 (*n* = 3 independent samples/group)

To elucidate the role of UDP-glucose ceramide glucosyltransferase (UGCG) in regulating cerebral endothelial survival and angiogenesis, we first evaluated the effects of the pharmacological inhibitor D-PDMP administered at two doses (25 or 50 µM) on cerebral microvascular endothelial cells (hCMEC/D3). LC–MS/MS revealed that UGCG deactivation by D-PDMP almost completely suppressed total hexosylceramide levels in cerebral microvascular endothelial cells (hCMEC/D3) at two examined doses (25 or 50 µM) (Fig. 1A). Hexosylceramide comprises the configuration isomers glucosylceramide and galactosylceramide, which could not be separated chromatographically using the technique applied and therefore could not be distinguished by LC–MS/MS due to their identical molecular mass. The strong reduction of total hexosylceramide levels was associated with marked decreases of several hexosylceramide species, namely C16, C18, C20, C22, C24 and C24:1 hexosylceramides (Fig. 1B-G), which is consistent with the reported effect of D-PDMP [38]. In contrast, lactosylceramides, which are formed by galactosylation of glucosylceramides catalyzed by lactosylceramide synthase, were not reduced by D-PDMP treatment (Fig. 2A-G). Of note, D-PDMP-mediated UGCG inhibition at 50 µM, but not 25 µM significantly increased total ceramide and more specifically C16, C18, C20, C22, and C24:1 ceramide species (Fig. 3A-G). D-PDMP also significantly increased S1P levels at 50 µM (Fig. 3H). Sphingomyelin levels were not influenced by the UGCG inhibitor (Fig. 4A-G).

### Pharmacological UGCG Inhibition Increases Cerebral Angiogenesis *in vitro*, but at a High Dose Compromises Endothelial Survival

**Fig. 5:**
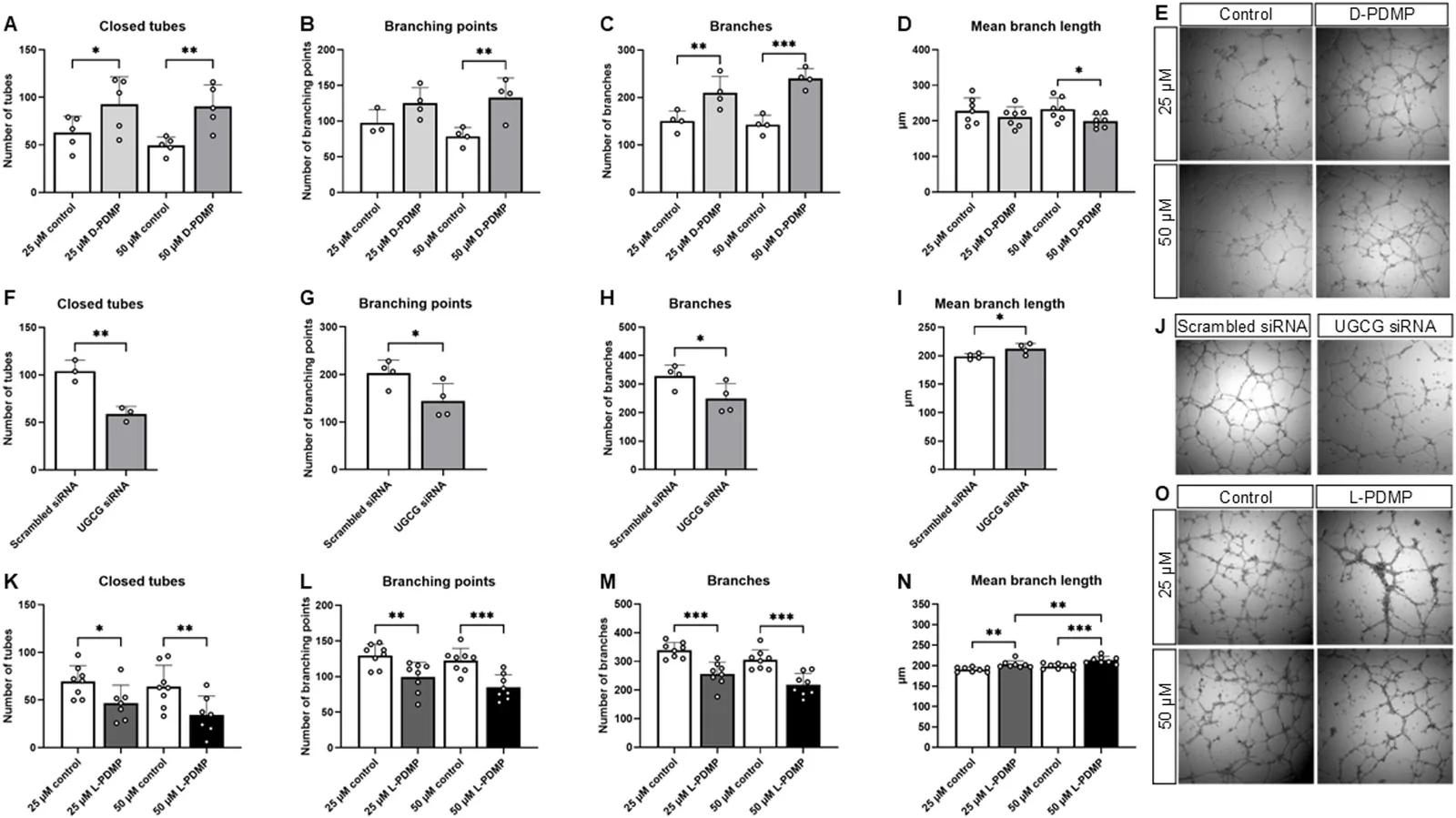
Pharmacological UGCG deactivation increases endothelial tube formation, whereas UGCG knockdown and UGCG activation inhibit it. hCMEC/D3 were seeded on matrigel and treated with vehicle, D-PDMP or L-PDMP (25 µM or 50 µM each) for 24 h, or were transfected with scrambled siRNA or UGCG siRNA. Tube formation was assessed by quantifying the number of closed tubes (**A, F, K**), number of branching points (**B, G, L**) number of branches (**C, H, M**) and mean branch length (**D, I, N**). Representative photographs are depicted in (**E, J, O**). Data are presented as mean + SD. Statistical analysis was performed using one-way ANOVA followed by LSD post hoc tests for the pharmacological UGCG deactivation and activation experiments, or two-tailed unpaired t-tests for the siRNA mediated knockdown experiments. Statistical significance is indicated as *p < 0.05, **p < 0.01 or ***p < 0.001 (*n* = 5 independent samples/group in (**A**), *n* = 3–4 independent samples/group in (**B**), *n* = 3 independent samples/group in (**F**), *n* = 4 independent samples/group in (**C**, **G**-**I**), *n* = 7–8 independent samples/group in (**D**, **K**-**N**))

**Fig. 6:**
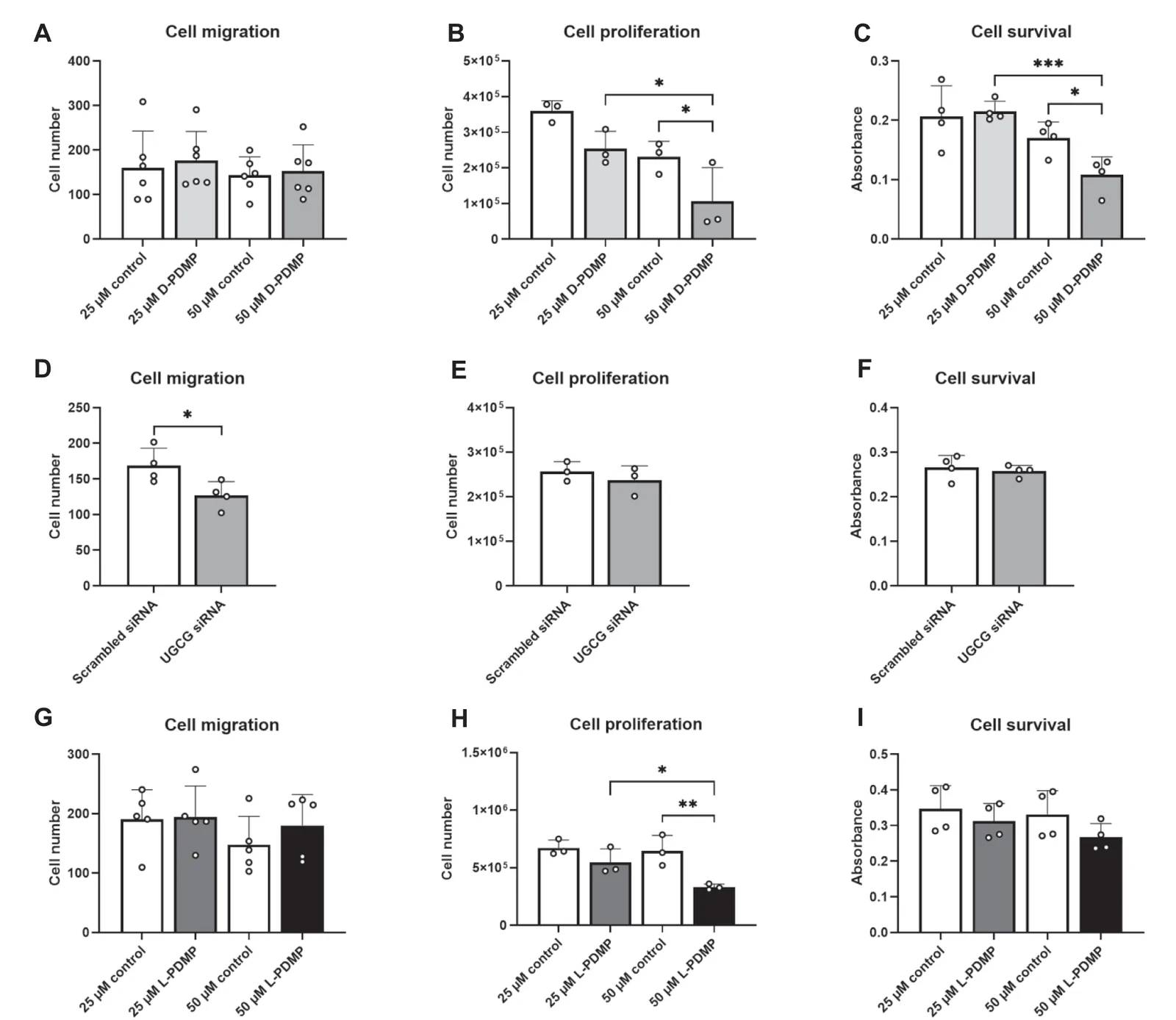
UGCG deactivation, but not UGCG knockdown reduces endothelial proliferation and survival, while UGCG activation reduces endothelial proliferation. Transwell migration (**A, D, G**), cell proliferation (**B, E, H**) and cell survival (**C, F, I**) were assessed in hCMEC/D3 treated with vehicle, D-PDMP or L-PDMP (25 µM or 50 µM each) for 24 h, or were transfected with scrambled siRNA or UGCG siRNA. Data are presented as mean + SD. Statistical analysis was performed using one-way ANOVA followed by LSD post hoc tests for the pharmacological UGCG deactivation and activation experiments, or two-tailed unpaired t-tests for the siRNA mediated knockdown experiments. Statistical significance is indicated as *p < 0.05, **p < 0.01 or ***p < 0.001 (*n* = 6 independent samples/group in (**A**), *n* = 3 independent samples/group in (**B**, **E**, **H**), *n* = 4 independent samples/group in (**C**, **D**, **F**, **I**), *n* = 5 independent samples/group in (**G**))

To elucidate the functional consequences of UGCG inhibition, we examined the effects of D-PDMP on cerebral endothelial survival and angiogenesis of hCMEC/D3 using matrigel-based tube formation, transwell-migration, proliferation and survival assays. In tube formation assays, D-PDMP administered at 25 or 50 µM significantly increased the number of closed capillary-like tubes (Fig. 5A, E), branching points (Fig. 5B, E) and branches (Fig. 5C, E), and, when administered at 50 µM, significantly reduced mean branch length (Fig. 5D, E). Endothelial migration was not affected by the UGCG inhibitor (Fig. 6A), whereas endothelial proliferation and survival were significantly reduced by D-PDMP at 50 µM, but not 25 µM (Fig. 6B, C).

### siRNA-Mediated UGCG Knockdown More Modestly Reduces Hexosylceramide Levels and Reduces S1P Levels in Cerebral Endothelial Cells

**Fig. 7:**
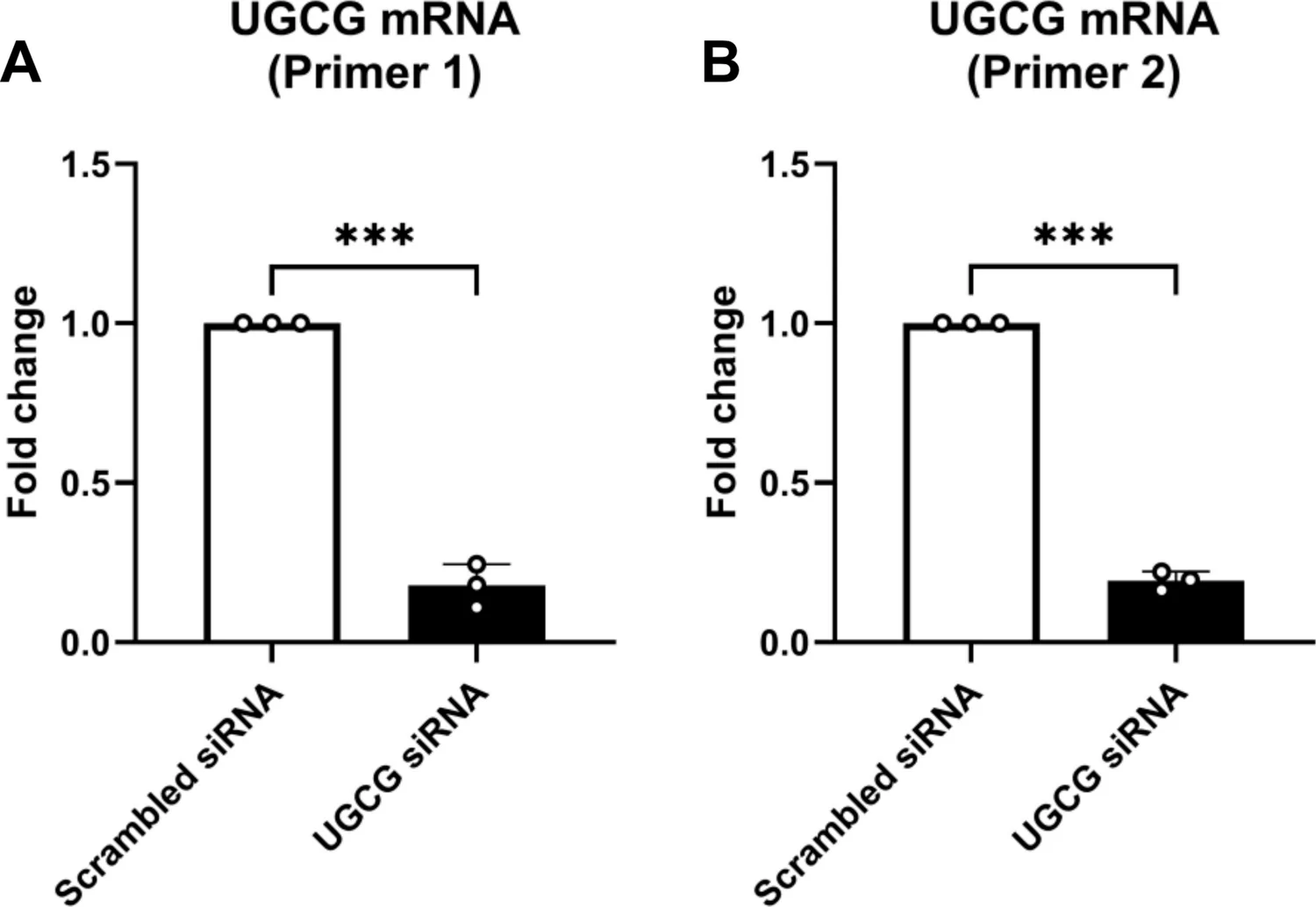
UGCG knockdown can be achieved by small interfering RNA (siRNA) *in vitro*. Successful downregulation of UGCG gene expression was confirmed by RT-qPCR using two different primers in hCMEC/D3 transfected with scrambled siRNA or UGCG siRNA (**A, B**). Data are means + SD values. Statistical analysis was performed by two-tailed unpaired t tests. Statistical significance is indicated as ***p < 0.001 (*n* = 3 independent samples/group in (**A**, **B**))

To substantiate the specificity of the pharmacological UGCG inhibition experiments, we chose a second strategy and selectively reduced UGCG expression by siRNA in hCMEC/D3. Successful knockdown was confirmed by PCR at the mRNA level (UGCG mRNA reduction by 82.23% ± 6.73% (Primer 1) and 80.7% ± 2.91% (Primer 2) compared with scrambled siRNA) (Fig. 7A, B). Sphingolipid profiling by LC–MS/MS revealed that UGCG knockdown more moderately reduced hexosylceramide levels, which was significant for C16 and C18 hexosylceramides (Fig. 1), but did not influence lactosylceramide (Fig. 2), ceramide (Fig. 3), or sphingomyelin (Fig. 4) levels, except for C24 sphingomyelin, which was increased after UGCG knockdown (Fig. 4F). Strikingly, in contrast to pharmacological UGCG deactivation, UGCG knockdown significantly reduced S1P levels (Fig. 3H).

### UGCG Knockdown Decreases Cerebral Angiogenesis *in vitro*

Probably as a result of the different sphingolipid pattern, UGCG knockdown did not recapitulate the pro-angiogenic effects of pharmacological UGCG deactivation, but significantly decreased the number of closed capillary-like tubes (Fig. 5F, J), branching points (Fig. 5G, J) and branches (Fig. 5H, J), and significantly increased mean branch length (Fig. 5I, J) of hCMEC/D3 in tube formation assays. Endothelial migration was reduced by UGCG knockdown (Fig. 6D), whereas endothelial proliferation and survival were not influenced by UGCG knockdown (Fig. 6E, F).

### Pharmacological UGCG Activation Increases Hexosylceramide Levels Without Influencing Ceramides and S1P in Cerebral Endothelial Cells

We next explored how pharmacological UGCG activation by L-PDMP influenced cerebral endothelial survival and angiogenesis of hCMEC/D3. LC–MS/MS revealed that the UGCG activator L-PDMP significantly increased C16 and C18 hexosylceramide levels of hCMEC/D3, when administered at 50 µM, but not 25 µM (Fig. 1A-G). As before, lactosylceramide levels were not influenced by L-PDMP (Fig. 2A-G), and ceramides and S1P were only tendentially increased (Fig. 3A-H). Sphingomyelin levels were not altered by UGCG activation, with the exception of C24:1 sphingomyelin, which was reduced by high dose (50 µM) L-PDMP (Fig. 4A-G).

### UGCG Activation Decreases Cerebral Angiogenesis *in vitro*

To examine the functional consequences of UGCG activation, we also explored the effect of L-PDMP on cerebral angiogenesis and endothelial survival of hCMEC/D3 *in vitro*. In tube formation assays, L-PDMP administered at 25 or 50 µM significantly reduced the number of closed capillary-like tubes (Fig. 5K, O), branching points (Fig. 5L, O) and branches (Fig. 5M, O), and furthermore, increased mean branch length (Fig. 5N, O). Endothelial migration and survival were not affected by the UGCG activator (Fig. 6G, I), but endothelial proliferation was significantly reduced by treatment with L-PDMP at 50 µM (Fig. 6H). These data reveal that UGCG activation negatively impacts cerebral angiogenesis in vitro.

### Pharmacological Modulation of UGCG Promotes Small EV Release of Cerebral Endothelial Cells, which Confer Anti-Angiogenic Activities

**Fig. 8:**
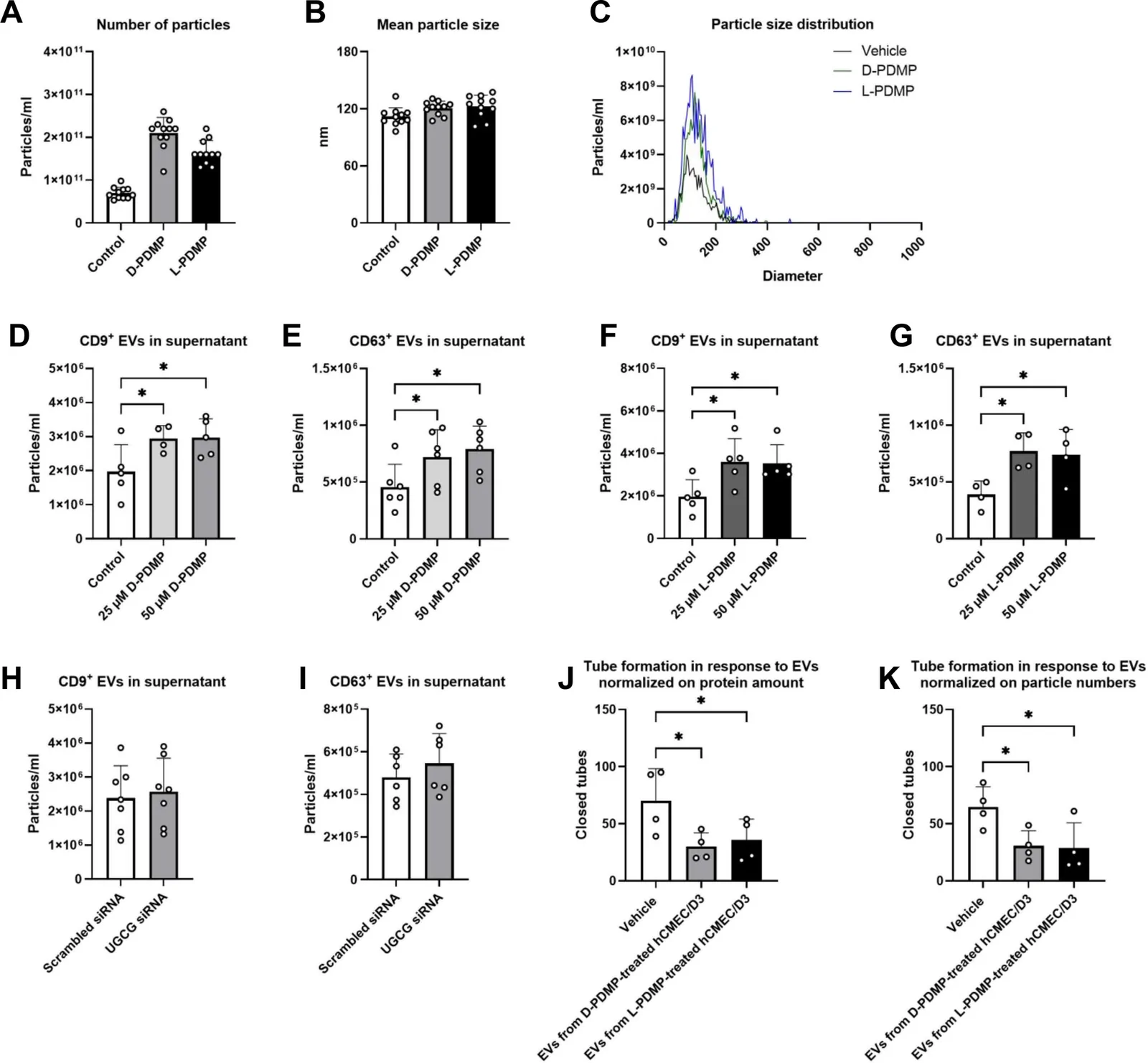
Pharmacological modulation of UGCG, but not UGCG knockdown, increases the release of EVs with anti-angiogenic activity. EVs were isolated from vehicle-, D-PDMP (50 µM)- or L-PDMP (50µM)-treated cells and the EV particle number, mean EV size and particle size distribution were assessed by nanoparticle tracking analysis (NTA) (**A-C**). The concentration of CD9^+^ or CD63^+^ sEVs was evaluated by AMNIS flow cytometry in the supernatant of hCMEC/D3 treated with vehicle, D-PDMP or L-PDMP (25 µM or 50 µM each) for 24 h, or transfected with scrambled siRNA or UGCG siRNA (**D-I**). Matrigel-based tube formation assay was conducted for hCMEC/D3 that were treated with vehicle or EVs, that were normalized on protein amounts (**J**, 25 µg/ml) or particle numbers (**K**, 1 × 10^8^ particles/well) and were isolated from D-PDMP (50 µM) or L-PDMP (50 µM) treated cells. Data are presented as mean + SD. Statistical analysis was performed using one-way ANOVA followed by LSD post hoc tests for the pharmacological UGCG deactivation and activation experiments, or two-tailed unpaired t-tests for the siRNA mediated knockdown experiments. Statistical significance is indicated as *p < 0.05 (*n* = 11 technical replicates in (**A**, **B**), *n* = 4–5 independent samples/group in (**D**, **F**, **G**, **J**, **K**), *n* = 6 independent samples/group in (**E**, **I**), *n* = 7 independent samples/group in (**H**))

Since sphingolipids critically influence membrane fluidity and fusogenicity and control the release of small EVs [31, 42], which are important mediators of intercellular communication [16], we investigated how D-PDMP mediated UGCG inhibition or L-PDMP mediated activation affect EV secretion of hCMEC/D3. Following EV isolation, nanoparticle tracking analysis (NTA) revealed that particles yields were increased in both cases compared to the vehicle group (7 × 10^10^ ± 1.4 × 10^10^), with 2.2 × 10^11^ ± 2.2 × 10^10^ particles measured after D-PDMP treatment and 1.7 × 10^11^ ± 2.8 × 10^10^ particles following L-PDMP treatment (Fig. 8A). The average particle size determined by NTA was 118.8 ± 9.3 nm in the vehicle group, 120.3 ± 7.6 nm after D-PDMP treatment and 122.4 ± 11.9 nm following L-PDMP treatment (Fig. 8B, C). For EV analysis, we performed single EV analysis on the AMNIS Image Stream RX platform. Consistent with the NTA data, we observed higher numbers of CD9^+^ and CD63^+^ vesicles for each of the two D-PDMP and L-PDMP doses (25 µM and 50 µM) (Fig. 8D-G).

Since pharmacological UGCG inhibition induced a different sphingolipid pattern compared to siRNA mediated UGCG knockdown, we also explored the small EV content following UGCG knockdown. Here, in contrast to UGCG inhibition by D-PDMP treatment, EV release was not significantly influenced (Fig. 8H, I). To test their cell biological activities, small EVs were harvested from conditioned media and applied to hCMEC/D3. Notably, EVs obtained from D-PDMP and L-PDMP treated cells exhibited potent anti-angiogenic activity in tube formation assays (Fig. 8J, K).

### Pharmacological UGCG Inhibition and Activation Induce Microvascular Network Degeneration After Ischemic Stroke *in vivo*

**Fig. 9:**
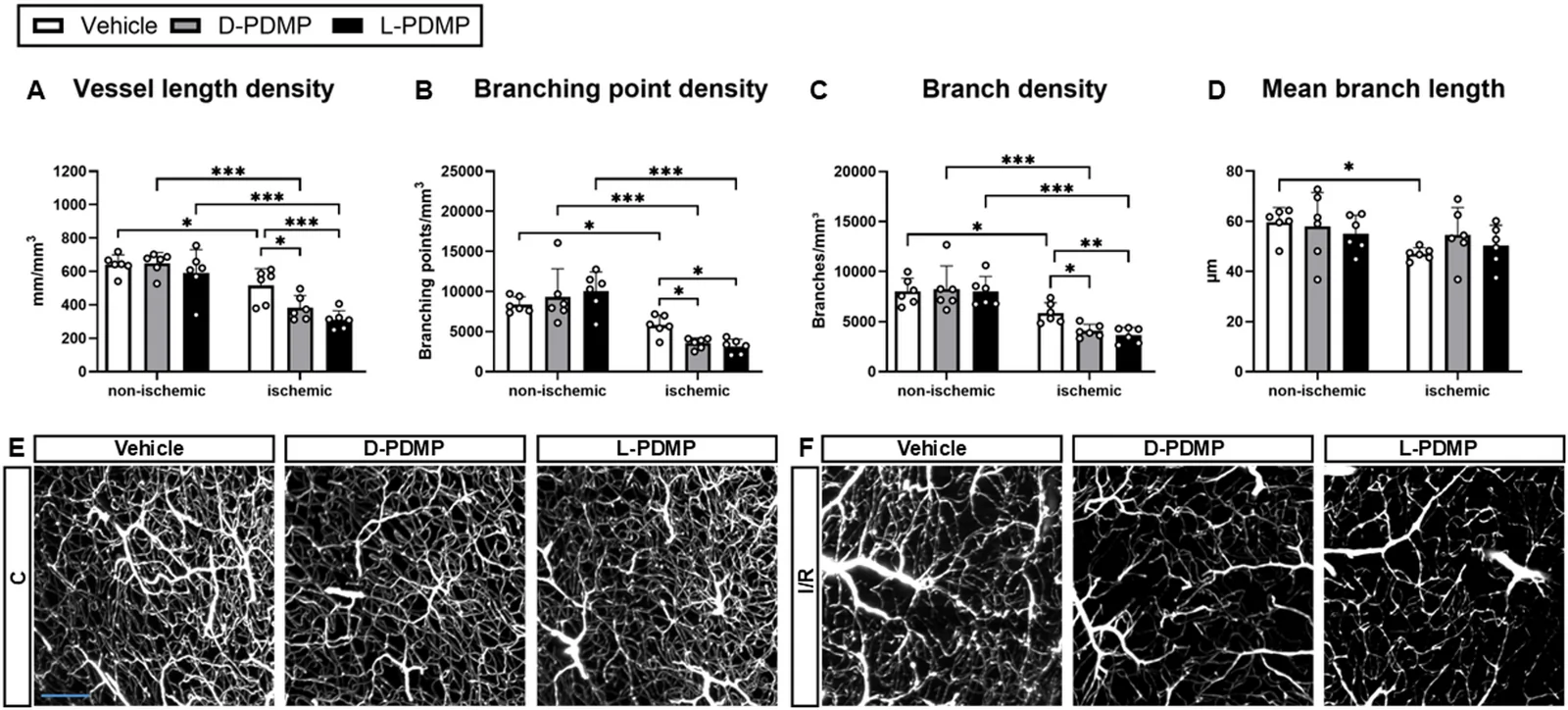
Pharmacological modulation of UGCG induces microvascular network degeneration after ischemic stroke in mice. Microvascular length density (**A**), branching point density (**B**), branch density (**C**) and mean microvascular branch length (**D**) were evaluated by light sheet fluorescence microscopy (LSFM) in the ischemic and contralesional non-ischemic striatum of mice exposed to transient middle cerebral artery occlusion (MCAO) followed by treatment with vehicle, D-PDMP (40 mg/kg, i.p.) or L-PDMP (40 mg/kg, i.p.) starting 24 h until 14 days after MCAO. Representative maximum projection images of the contralesional non-ischemic striatum (labeled C) and in the reperfused ischemic striatum (labeled I/R) are shown in (**E**) and (**F**). Data are means + SD values. Statistical analysis was performed using two-way ANOVA followed by LSD post hoc tests. Statistical significance is indicated as *p < 0.05, **p < 0.01 or ***p < 0.001 (*n* = 6 mice/group)

**Fig. 10:**
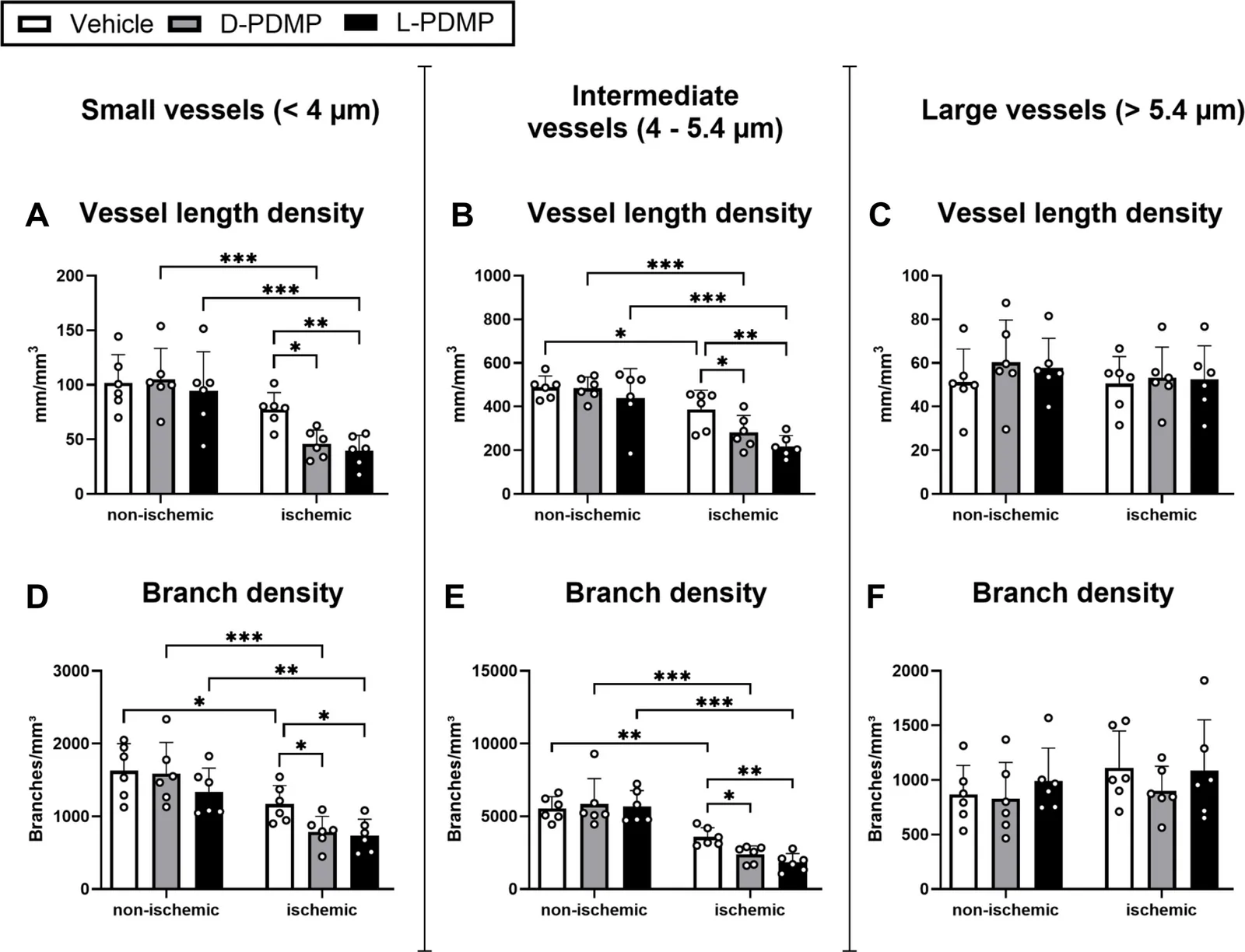
Pharmacological modulation of UGCG induces microvascular degeneration of small and intermediate-sized vessels after ischemic stroke. Microvessels were discriminated according to their diameter. The vessel length density of (**A**) small diameter (< 4 µm), (**B**) intermediate diameter (4–5.4 µm) and (**C**) large diameter (> 5.4 µm) microvessels, as well as the branch density of (**D**) small diameter (< 4 µm), (**E**) intermediate diameter (4–5.4 µm) and (**F**) large diameter (> 5.4 µm) microvessels were evaluated by LSFM in the ischemic and contralesional non-ischemic striatum of MCAO mice treated with vehicle, D-PDMP (40 mg/kg, i.p.) or L-PDMP (40 mg/kg, i.p.) starting 24 h until 14 days after MCAO. Data are means + SD values. Statistical analysis was performed using two-way ANOVA followed by LSD post hoc tests. Statistical significance is indicated as *p < 0.05, **p < 0.01 or ***p < 0.001 (*n* = 6 mice/group)

In order to investigate how UGCG activity influences cerebral microvascular integrity and angiogenesis *in vivo* after ischemic stroke, mice were subjected to transient MCAO and treated with vehicle, D-PDMP (40 mg/kg, i.p.) or L-PDMP (40 mg/kg, i.p.) for 14 days starting 24 h after reperfusion. This experimental design was selected to assess the effects of UGCG modulation during the post-ischemic vascular remodeling phase, as early ischemia-associated responses could otherwise confound the interpretation of remodeling-specific effects. Microvascular network remodeling was examined using the tracer FITC-albumin by 3D light-sheet microscopy, followed by an unbiased analysis of microvascular network characteristics using the automated software VesselExpress [37]. Microvessels were categorized by diameter as small (< 4 µm), intermediate (4–5.4 µm), or large (> 5.4 µm) microvessels. Both D-PDMP and L-PDMP induced cerebral microvascular degeneration in the previously ischemic striatum, as evidenced by a decrease of vessel length density, branching point density and branch density (Fig. 9A-C, E, F). Mean branch length in the previously ischemic hemisphere was not significantly influenced by D-PDMP and L-PDMP (Fig. 9D, E, F). Both small and intermediate diameter microvessels, which are most strongly damaged by I/R injury [10], [11], were reduced by D-PDMP and L-PDMP (Fig. 10A, B, D, E), while large diameter vessels were unaffected (Fig. 10C, F). At this time point, lesion volume and FITC-albumin extravasation as a measure of vascular integrity were not significantly influenced by pharmacological UGCG manipulation (Suppl. Figure 1B, C). Laser Doppler flowmetry measurements confirmed that the extent of occlusion and subsequent reperfusion did not differ significantly between experimental groups (Suppl. Figure 1 A), indicating that variations in cerebral blood flow were unlikely to have confounded the observed treatment effects.

## Discussion

We herein report that pharmacological UGCG deactivation by D-PDMP, which markedly reduced cellular hexosylceramide levels, but not siRNA-mediated UGCG knockdown, which more moderately decreased predominantly short (C16 and C18) hexosylceramide levels, promoted cerebral angiogenesis and at high dosage reduced cerebral microvascular endothelial survival *in vitro* via mechanisms that involved increased S1P and increased ceramide formation. In contrast, UGCG activation by L-PDMP, which increased hexosylceramide levels without affecting ceramide or S1P, reduced angiogenesis revealed by tube formation assays. Both D-PDMP and L-PDMP enhanced the release of EVs by cerebral endothelial cells that had anti-angiogenic activity. *In vivo*, in the MCAO model, both D-PDMP and L-PDMP similarly induced microvascular network degeneration in the reperfused striatum. Our study specifically focused on the post-ischemic remodeling phase, as the first 24 h after ischemia are characterized by dynamic sphingolipid remodeling, including ceramide accumulation and its potential conversion into downstream sphingolipid species [31]. These acute metabolic changes may contribute to early injury responses and therefore, treatment was initiated after 24 h of reperfusion to specifically investigate the role of UGCG modulation during the subsequent vascular remodeling phase. Our findings fundamentally differ from previous studies in neurons, in which pharmacological UGCG activation by L-PDMP promoted neuronal survival and enhanced synaptic plasticity *in vitro*, and findings in focal and global cerebral ischemic models in rats, in which UGCG activation in the post-acute phase (also starting 24 h post-I/R) enhanced learning and memory deficits [17, 20]. Conversely, UGCG deactivation by D-PDMP impaired neuronal survival, disturbed calcium homeostasis, and decreased synaptic plasticity in earlier studies [21, 45].

Pharmacological UGCG deactivation *in vitro* increased cerebral endothelial S1P levels, which is a well-characterized bioactive lipid that promotes cytoskeletal reorganization, cell–cell junction integrity, and nitric oxide production, all crucial for microvascular integrity and angiogenic sprouting [19, 22]. Increased S1P formation after pharmacological UGCG inhibition has previously been reported in histiocytic lymphoma cells [4], and was attributed to ceramide accumulation, which was converted to sphingosine (by ceramidases) and phosphorylated to form S1P (by sphingosine kinases). While S1P supports angiogenic activity, this effect is fragile as the simultaneous and sustained accumulation of ceramide, which is associated with pro-apoptotic signaling and microvascular degeneration [31], undermines endothelial survival, as we furthermore observed *in vitro*. In addition to its role in endothelial angiogenic signaling, S1P is a multifunctional lipid mediator involved in several aspects of the post-ischemic response [29]. Previous studies have demonstrated that S1P signaling influences vascular stability and neurological outcome, while pharmacological modulation of S1P signaling can affect immune infiltration and microvascular remodeling after stroke.

[11, 14, 18, 23, 27, 32, 44]. The effects of S1P on vascular tone depend on the cellular context and receptor subtype activated. Activation of endothelial S1PR1 for instance can enhance nitric oxide production through eNOS signaling and promote vasorelaxation [18]. Therefore, the increase in endothelial S1P observed following UGCG inhibition may have broader effects beyond angiogenic sprouting. However, the present study focused on structural aspects of microvascular remodeling and did not directly assess the contribution of altered S1P signaling to vascular tone, immune responses, or cerebral perfusion. Notably, siRNA-mediated knockdown of UGCG more moderately reduced hexosylceramide levels, and, unlike D-PDMP, did not increase, but reduced S1P and impaired angiogenesis. While D-PDMP is commonly used as a UGCG inhibitor, it has been reported to induce lysosomal accumulation of lipids, including sphingolipids, lysobisphosphatidic acid and cholesterol, which were attributed to defective lysosomal function and export [13]. The preferential lysosomal accumulation of D-PDMP may explain the differential effect on sphingolipids compared with genetic UGCG knockdown. In addition, the more moderate effect of siRNA-mediated UGCG knockdown on hexosylceramide levels may be influenced by residual UGCG protein abundance or activity, as well as compensatory regulation within the sphingolipid metabolic pathway. siRNA-mediated UGCG knockdown further increased C24 sphingomyelin, a very-long-chain species known to distinctively increase membrane rigidity [7]. In contrast to pharmacological UGCG deactivation, UGCG activation by L-PDMP did not increase S1P and reduced angiogenesis. Further, L-PDMP selectively reduced C24:1 sphingomyelin, which critically maintains membrane fluidity [7]. This specific loss of a fluidizing acyl chain likely alters microdomain organization and downstream signaling. Of note, L-PDMP-induced accumulation of glycosylated ceramide mimics the pathological sequelae of Gaucher disease, in which hydrolysis of glucosylceramide to ceramide and glucose is impaired, fostering the accumulation of glucosylceramide and its derivative glucosylsphingosine in lysosomes [40]. In a neuronopathic Gaucher disease mouse model, elevated glycosphingolipids were associated with reduced cerebral microvascular density and impaired endothelial kinesis [35], confirming the observations of this study *in vitro* and *in vivo* that excess glycosylated ceramides adversely affect microvascular network integrity.

Interestingly, in our study both PDMP enantiomers promoted the release of small EVs by cerebral endothelial cells, which reduced angiogenesis *in vitro*. Future studies assessing circulating or brain-derived EVs will be important to determine their potential contribution to post-ischemic microvascular remodeling *in vivo*. Glycosylated ceramides crucially define cellular membrane properties, more specifically membrane interactions with hydrophilic environments in late endosomes, where small EVs (i.e., exosomes) are formed. From this perspective, it is not surprising that EV formation was altered both by excess and reduced glycosylated ceramide formation. Of note, siRNA-mediated UGCG knockdown, which more moderately influenced hexosylceramide levels, did not influence cerebral endothelial small EV secretion. This finding suggests that a certain level of hexosylceramide changes is needed for small EVs to be released. Alternatively, late endosomal and lysosomal specific effects of D-PDMP and L-PDMP must be considered, which increased EV release by endothelial cells. In a previous study, D-PDMP induced lysosomal lysobisphosphatidic acid enrichment was associated with mechanistic target of rapamycin (mTOR) deactivation [13]. Given that active mTOR complex 1 (mTORC1) has been reported to suppress EV release via a Rab27A‐dependent mechanism [47], it is conceivable that mTOR deactivation may contribute to enhanced vesicle secretion, providing a plausible mechanistic link between sphingolipid disruption, lysosomal stress, and EV biogenesis. In prostatic adenocarcinoma cells, the ceramide glucosyltransferase inhibitor PDMP has previously been shown to change the composition of secreted EVs, elevating their caveolin-1 and annexin A2 abundance [33]. Caveolin-1 and annexin A2 are lipid raft associated proteins responsible for the recruitment of cargo, including extracellular matrix and miRNA, into EVs [1, 12]. The altered EV cargo may contribute to the anti-angiogenic properties of EVs, although the specific components responsible for this effect remain to be defined. It is important to note that dissecting off-target effects of pharmacological UGCG inhibitors or activators *in vivo* effects remains challenging, as global UGCG knockout in mice is embryonically lethal [46]. Future studies will have to clarify these questions using conditional or tissue-specific knockout mice. In addition, further investigation is warranted to determine whether systemic D-PDMP and L-PDMP treatment alters ceramide, S1P, and hexosylceramide levels in the ischemic brain or cerebral microvessels. Vascular repair is a complex process involving multiple components of the neurovascular unit. Therefore, additional characterization of pericyte–endothelial interactions, and endothelial cell survival, could further clarify the contribution of distinct neurovascular components to UGCG-mediated effects after stroke. A limitation of this study is that all *in vivo* experiments were performed in young adult male mice. Sex and age are important biological variables in stroke pathophysiology and may influence ischemic injury, vascular responses, and treatment outcomes. Therefore, future studies including female animals and different age groups will be required to determine the extent to which these findings are generalizable across sexes and different age groups.

## Conclusion

While the neuronal plasticity-promoting effects of pharmacological UGCG activation *in vitro* and *in vivo* encouraged the use of UGCG activators for enhancing neurological recovery post-ischemic stroke [17, 20], both pharmacological UGCG deactivation and activation impaired microvascular network remodeling in a mouse MCAO model, resulting in the degeneration of cerebral microvessels via mechanisms involving the release of anti-angiogenic small EVs. The detrimental effects on cerebral microvascular integrity and angiogenesis challenges the use of UGCG activators in I/R injuries.

## Supplementary Information

Below is the link to the electronic supplementary material.

Supplementary file1 Pharmacological modulation of UGCG does not influence lesion volume or FITC-albumin extravasation at day 14 after ischemic stroke. (**A**) Laser Doppler flow (LDF) was monitored during surgery in mice subjected to 30-min middle cerebral artery occlusion (MCAO). (**B**) Lesion volume was assessed after 14 days of reperfusion based on the 561-nm autofluorescence signal. (**C**) Extravascular FITC fluorescence was quantified after 14 days of reperfusion and normalized to vessel area. Representative images showing FITC-albumin fluorescence and threshold-based segmentation of intravascular FITC signal. Data are means + SD values. Statistical analysis was performed using two-way repeated-measures ANOVA followed by LSD post hoc test for LDF recordings. Lesion volumes and FITC-albumin extravasation were analyzed using one-way ANOVA followed by LSD post hoc test (n=6 mice/group). (PDF 175 KB)

## Data Availability

The data that support the findings of this study are available from the corresponding author upon reasonable request.

## Declarations

### Research Involving Humans and Animals Statement

This article does not contain any studies with human participants. All applicable international, national and institutional guidelines for the care and use of animals were followed.
