# Supplementary material for: Roles of Ceramide Glucosyltransferase in Controlling Cerebral Microvascular Endothelial Integrity and Angiogenesis Post-Ischemia/Reperfusion
Source: Transl Stroke Res. 2026 Aug 21;17(5):98. doi: 10.1007/s12975-026-01488-9 (PMC13493440; doi:10.1007/s12975-026-01488-9)

Suppl. Fig. 1: Pharmacological modulation of UGCG does not influence lesion volume or FITC-albumin extravasation at day 14 after ischemic stroke.

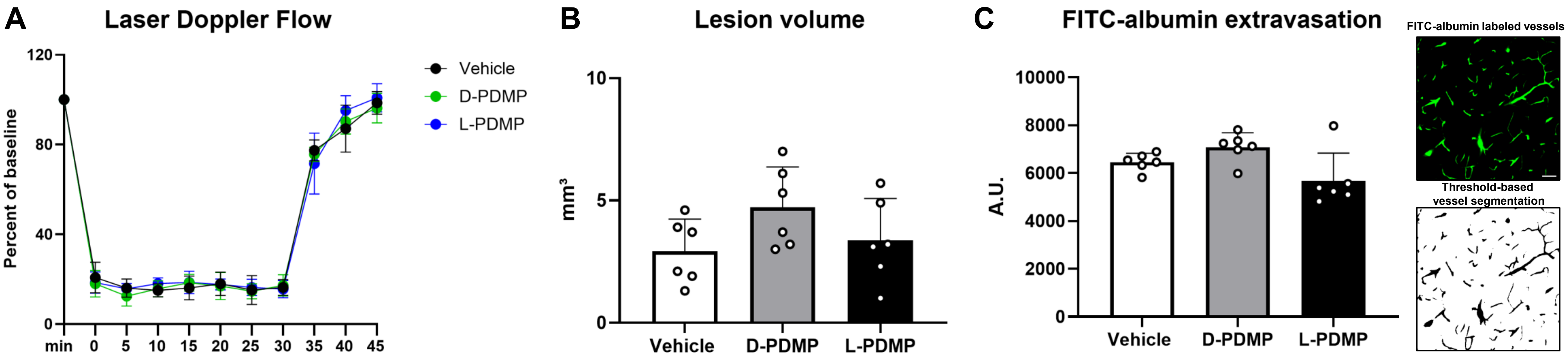

Supplement: Supplementary file 1 [file 12975_2026_1488_MOESM1_ESM.pdf]
